# PREPRINT Machine Learning for the Sensitivity Analysis of a Model of the Cellular Uptake of Nanoparticles for the Treatment of Cancer

**DOI:** 10.1002/cnm.3878

**Published:** 2024-10-29

**Authors:** Sarah Iaquinta, Shahram Khazaie, Samer Albanna, Sylvain Fréour, Frédéric Jacquemin

**Affiliations:** ^1^ Nantes Université, École Centrale Nantes, CNRS, GeM, UMR 6183 Saint‐Nazaire France

**Keywords:** adhesion, cancer cells, cellular uptake, machine learning, sensitivity analysis

## Abstract

Experimental studies on the cellular uptake of nanoparticles (NPs), useful for the investigation of NP‐based drug delivery systems, are often difficult to interpret due to the large number of parameters that can contribute to the phenomenon. It is therefore of great interest to identify insignificant parameters to reduce the number of variables used for the design of experiments. In this work, a model of the wrapping of elliptical NPs by the cell membrane is used to compare the influence of the aspect ratio of the NP, the membrane tension, the NP–membrane adhesion, and its variation during the interaction with the NP on the equilibrium state of the wrapping process. Several surrogate models, such as Kriging, Polynomial Chaos Expansion (PCE), and artificial neural networks (ANN) have been built and compared to emulate the computationally expensive model. Only the ANN‐based model outperformed the other approaches by providing much better predictivity metrics and could therefore be used to compute the sensitivity indices. Our results showed that the NP's aspect ratio, the initial NP–membrane adhesion, the membrane tension, and the delay for the increase of the NP–membrane adhesion after receptor dynamics are the main contributors to the cellular internalization of the NP, while the influence of other parameters is negligible.

## Graphical abstract

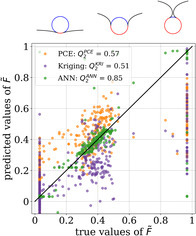

**Abbreviations**
ANA: antinuclear antibodies
APC: antigen‐presenting cells
IRF: interferon regulatory factor.

## 1 Presentation of the Model

To determine the properties that would favor their uptake by cancer cells based on a model, it is essential to accurately represent the mechanical behavior of the cell and its interaction with the NP. Thus, we developed a model of the cellular uptake of NPs that identifies the equilibrium position of the NP–membrane system based on the minimization of the potential energy, as detailed in [1]. This approach models the cell membrane as purely elastic, in alignment with existing literature [2, 3, 4], and treats the NP as an undeformable elliptic solid. The last assumption simplifies the model and is consistent with the characteristics of existing nanovectors, as discussed in [5]. The model consists of identifying the equilibrium position of the NP during its wrapping by the membrane via minimization of the total potential energy of the NP–membrane system. The membrane's mechanical properties are represented through its bending rigidity κ and tension σ. The NP's geometry is by its aspect ratio $\bar{r}$, defined as the ratio of the semi‐major axis to the semi‐minor axis, with a constant circumference 200π nm, equivalent to a circular NP with a radius a= 100 nm. The interaction between the NP and the membrane is modeled via the adhesion γ. To minimize the number of input parameters and to facilitate comparison with similar studies [6], adimensional variables are introduced: $\bar{\gamma }=\gamma {\left(2\kappa a^{2}\right)}^{-1}$ and $\bar{\sigma }=\sigma {\left(2\kappa a^{2}\right)}^{-1}$. In this article, a bar is placed over a quantity to indicate that it is adimensional. The NP's wrapping by the cell membrane is quantified by the wrapping degree f, representing the proportion of the NP's circumference enveloped by the membrane, varying between 0 and 1. The equilibrium state is defined by the wrapping degree at equilibrium $\tilde{f}$. Furthermore, this model, elaborated in [7], incorporates the membrane's mechano‐adaptation during the wrapping, allowing for the variation of $\bar{\gamma }$ as a function of f. The evolution model of $\bar{\gamma }$ during the wrapping process reads

(1) $$\bar{\gamma }\left(f\right)=\frac{|{\bar{\gamma }}_{0}\left({\bar{\gamma }}_{A}-1\right)|}{1+e^{-{\bar{\gamma }}_{S}\left(f-{\bar{\gamma }}_{D}\right)}}+{\bar{\gamma }}_{0},$$

wherein ${\bar{\gamma }}_{0}$ stands for the adhesion at the onset of the wrapping process, that is, ${\bar{\gamma }}_{0}=\bar{\gamma }\left(f=0\right)$. ${\bar{\gamma }}_{A}$ defines the amplitude of $\bar{\gamma }$, which corresponds to ${\bar{\gamma }}_{A}=\bar{\gamma }\left(f=1\right)/{\bar{\gamma }}_{0}$. ${\bar{\gamma }}_{D}$ denotes the delay before the inflexion of $\bar{\gamma }$ occurs, and ${\bar{\gamma }}_{S}$ is a parameter that modulates the slope of $\bar{\gamma }\left(f\right)$ during the transition. For a comprehensive mathematical description of these parameters together with graphical illustrations of their contribution to $\bar{\gamma }\left(f\right)$, the readers are referred to [7]. A summary of abbreviations and definitions of parameters is provided in the nomenclature section at the end of this article.

## 2 Surrogate Modeling and Deep Learning Approaches

### 2.1 Definition of the Input Dataset

Based on our previous studies [1, 7], the intervals of variation of the input parameters are defined according to the literature and some mathematical constraints as follows: ${\bar{\gamma }}_{0}\in \left[1,8\right]$, $\bar{\sigma }\in \left[0.5,5.5\right]$, ${\bar{\gamma }}_{A}\in \left[1,6\right]$, ${\bar{\gamma }}_{D}\in \left[-0.45,0.45\right]$, ${\bar{\gamma }}_{S}\in \left[10,100\right]$, and $\bar{r}\in \left[1/6,6\right]$. Since the only available information about the parameters is their lower and upper bounds, following the maximum entropy principle [8], their distribution is supposed to be uniform. Thus, ${\bar{\gamma }}_{0}$, $\bar{\sigma }$, ${\bar{\gamma }}_{A}$, ${\bar{\gamma }}_{D}$, and ${\bar{\gamma }}_{S}$ are modeled via random variables, defined as follows: ${\bar{\Gamma }}_{0}\sim U\left(1,8\right)$, $\bar{\sum }\sim U\left(0.5,5.5\right)$, ${\bar{\Gamma }}_{A}\sim U\left(1,6\right)$, ${\bar{\Gamma }}_{D}\sim U\left(-0.45,0.45\right)$, and ${\bar{\Gamma }}_{S}\sim U\left(10,100\right)$. The distribution of $\bar{R}$ is designed such that half of the dataset comprises horizontal NPs ($1\;<\;\bar{R}\;<\;6$), while the other half consists values of $\bar{R}$ that are the inverse of the aspect ratios of the horizontal ellipses. Therefore, the distribution of $\bar{R}$ when $\bar{R}\;<\;1$ should be the inverse of the uniform distribution that was used to maximize the entropy for the distribution of the horizontal NPs. The corresponding PDF is provided in [7].

**FIGURE 1:**
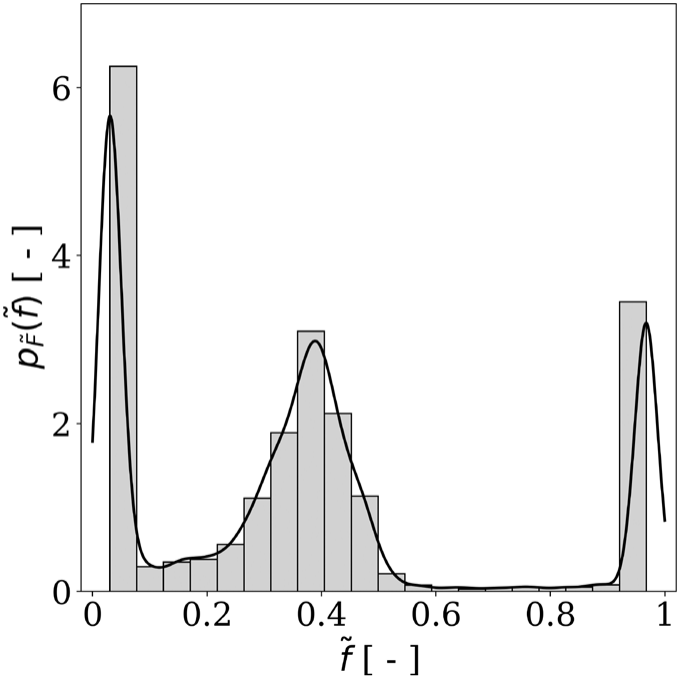
Histogram of $\tilde{F}$, the random variable associated to the wrapping degree at equilibrium $\tilde{f}$, based on the dataset, along with the kernel density estimation of its PDF.

After defining the distributions of the input parameters, a quasi‐Monte Carlo sampling method is utilized to generate the input dataset. The latter comprises $10^{12}=4096$ samples, representing the largest dataset that could be generated with reasonable computational cost. The output dataset contains the values of $\tilde{f}$ computed by the model for each realization of the input parameters. These values represent realizations of the random wrapping degree at equilibrium, denoted as $\tilde{F}$. The distribution of $\tilde{F}$ in the dataset is shown in Figure 1, along with a kernel density estimation of its probability density function (PDF). This dataset will be used to build the surrogate models in subsequent sections. The data exhibits a multimodal distribution with three peaks at $\tilde{f}\approx 0.03$, $\tilde{f}\approx 0.4$, and $\tilde{f}\approx 0.97$. The first peak can mainly be attributed to elongated vertical NPs, which require a significant and energy‐intensive bending of the membrane. This makes it challenging for the wrapping to overcome the close‐to‐zero wrapping degree at equilibrium. The peak of $\tilde{f}\approx 0.4$ arises similarly, but in this case, it is due to the wrapping of elongated horizontal NPs. For these NPs, wrapping one of the elongated sides to a degree of $\tilde{f}\approx 0.4$ does not require much energy, unlike the substantial bending needed to envelop the rest of the NP. The final peak at $\tilde{f}\approx 0.97$ pertains to cases where the wrapping is not significantly impeded by highly elongated NPs.

### 2.2 Kriging and Polynomial Chaos Expansion Metamodels

**FIGURE 2:**
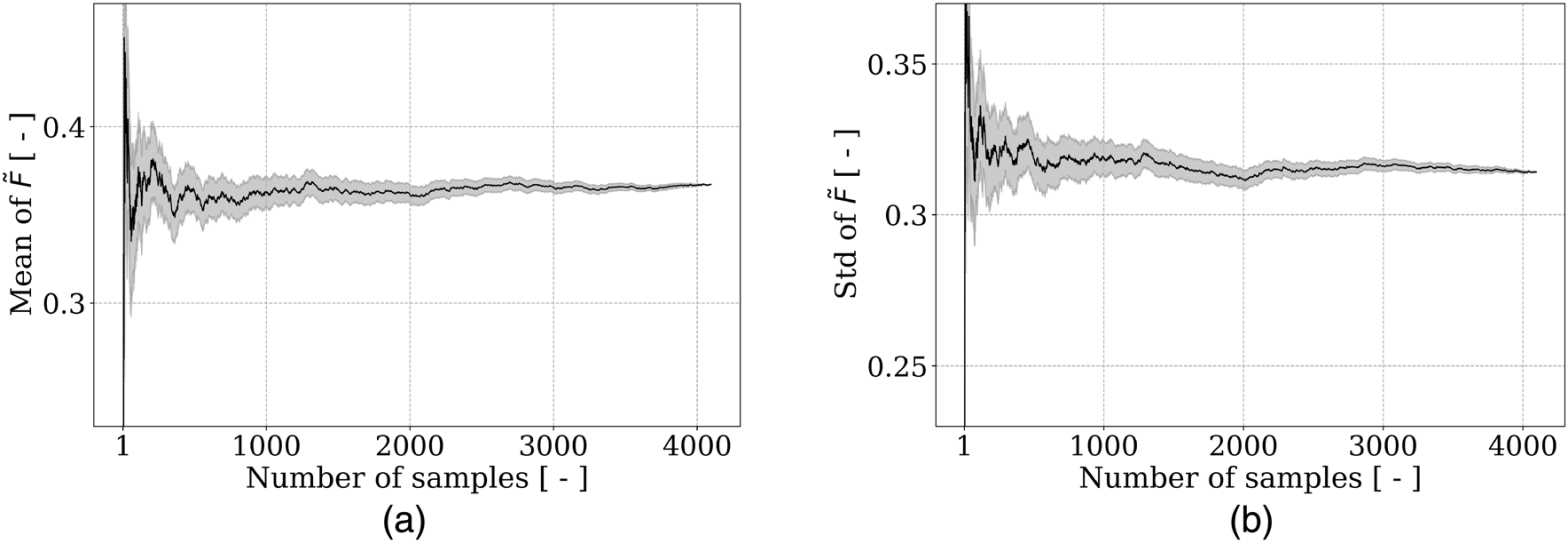
Variation of the (a) mean and (b) standard deviation of $\tilde{F}$, as a function of the number of simulations. The shaded regions correspond to the standard deviation, estimated using 200 shuffled samples.

**FIGURE 3:**
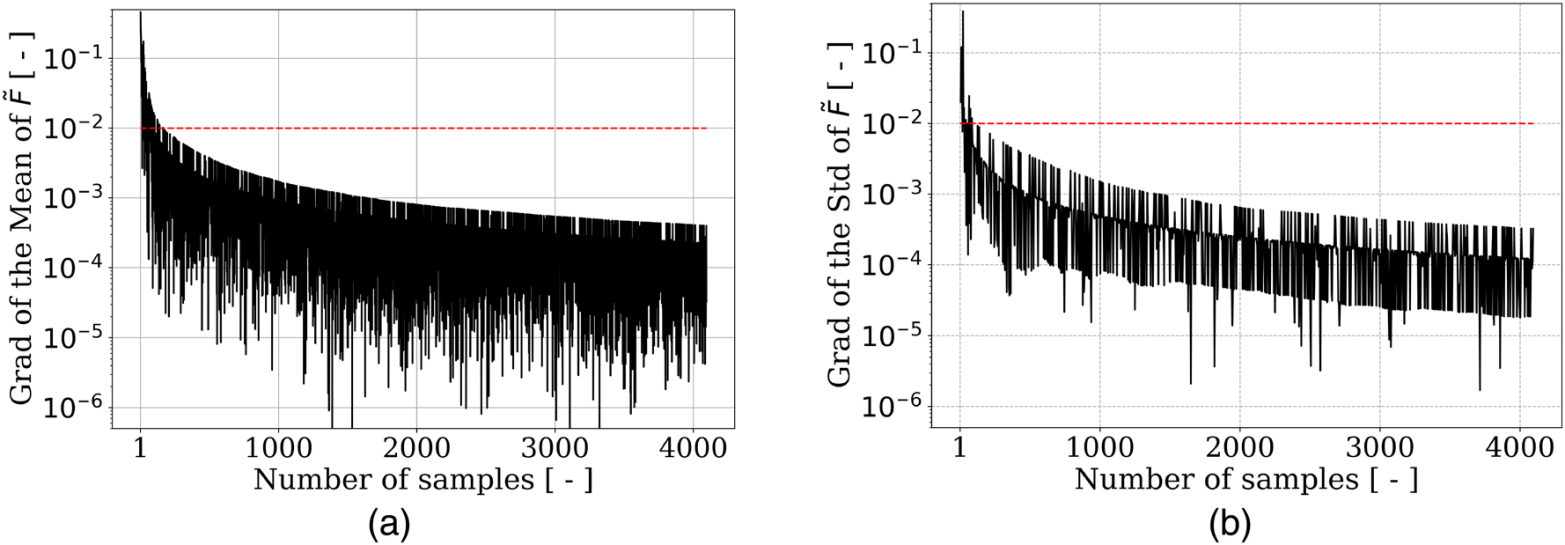
Normalized gradient of the (a) mean and (b) standard deviation of $\tilde{F}$, as a function of the number of simulations. The dashed lines correspond to the 1% threshold. Only one out of four points have been represented for the clarity of the plots.

This section compares the construction of Kriging (also known as Gaussian Process) and polynomial chaos expansion (PCE) metamodels. To determine the minimal number of samples that is necessary to be statistically representative of $\tilde{F}$, we first investigate the representativeness of the dataset. For this purpose, the cumulative mean and standard deviation of $\tilde{F}$, as well as their normalized gradient, in terms of the number of samples, are depicted in Figures 2 and 3, respectively. The normalized gradient of a function y with respect to a variable x is defined as $|y\left(x+1\right)-y\left(x\right)|/|y\left(x\right)|$. To ensure convergence (normalized gradient smaller than 1%), at least 158 and 122 samples are required for the mean and the standard deviation of $\tilde{F}$, respectively. Based on these results, 10% of the dataset (409 samples) will be used to validate the metamodels, and the remaining 90%, that is, 3687 samples, will be used for their training.

**FIGURE 4:**
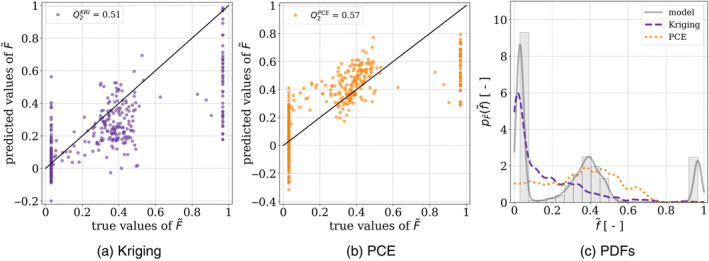
Predicted vs. true values obtained with (a) Kriging and (b) PCE algorithms after optimization of the hyperparameters, along with (c) a comparison of the PDFs obtained for these metamodels and that of the data from the model.

Both Kriging and PCE metamodels are built using the Open TURNS Python library, which enables the construction and postprocessing of surrogate models for sensitivity analyses [9]. Figure 4 displays the predictions and estimated PDF. The comparison between the estimated PDFs from the metamodel predictions and the original data reveals significant discrepancies, suggesting the surrogate model's inability to accurately replicate the data's distribution. Specifically, while the data exhibits a multimodal distribution with three peaks at $\tilde{f}\approx 0.03$, $\tilde{f}\approx 0.4$, and $\tilde{f}\approx 0.97$, the PCE predictions yield a unimodal distribution with a large peak around $\tilde{f}\approx 0.4$. In addition, the Kriging model results in a bimodal distribution with peaks located at $\tilde{f}\approx 0.03$ and $\tilde{f}\approx 0.25$. To further assess the accuracy of the metamodel predictions, we compute the predictivity factor $Q_{2}$ is computed. It is defined as

(2) $$Q_{2}=1-\frac{\sum_{i=1}^{N}{\left({\tilde{f}}_{i}-{\hat{\tilde{f}}}_{i}\right)}^{2}}{N\mathrm{Var}\left(\tilde{F}\right)},$$

in which ${\tilde{f}}_{i}$ stands for the $i^{\mathrm{th}}$ true value, ${\hat{\tilde{f}}}_{i}$ represents the corresponding predicted value, N indicates the size of the dataset, and Var is the variance. The predictivity factors of the PCE and Kriging models, denoted as $Q_{2}^{\mathrm{PCE}}$ and $Q_{2}^{\mathrm{KRI}}$ respectively, are unsatisfactory. Specifically, $Q_{2}^{\mathrm{KRI}}=0.51$ and $Q_{2}^{\mathrm{PCE}}=0.57$, values which significantly deviate from 1, indicating that the predictions lack accuracy. In this case, it appears that Kriging was unable to produce accurate estimations by interpolating the points in the dataset due to the dispersion of the data and a large number of input parameters that yielded equal values of $\tilde{F}$ in the training dataset. It is worth noting that prediction errors are concentrated around values of $\tilde{F}$ close to 0 and 1, which are the bounds of the domain of definition of the wrapping degree.

### 2.3 Artificial Neural Networks

**FIGURE 5:**
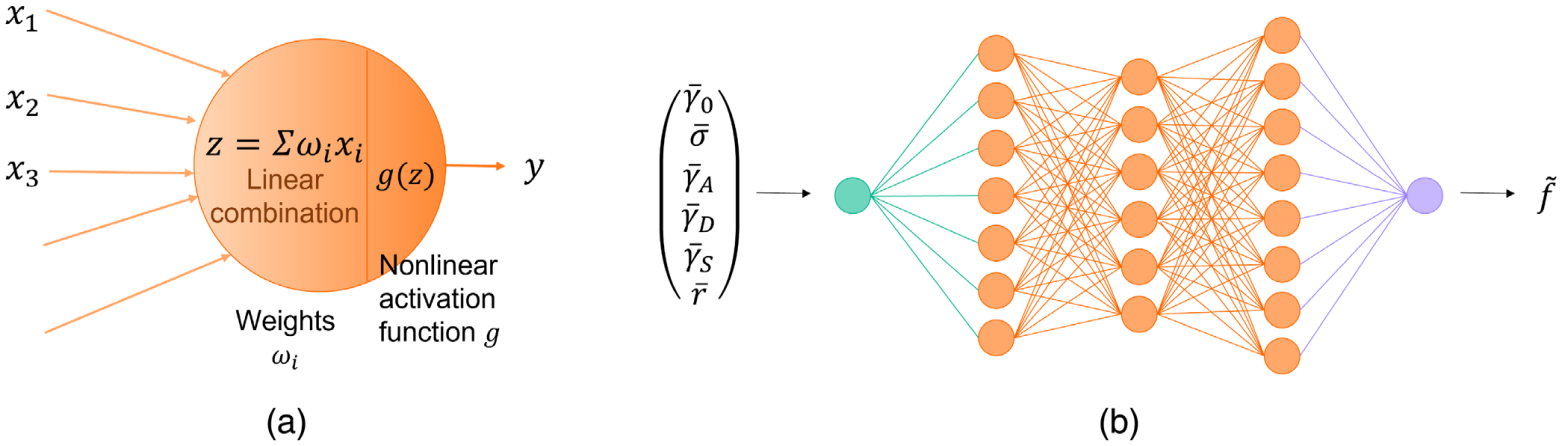
Illustration of (a) a neuron and (b) an artificial neural network.

Artificial neural networks (ANNs) are a type of algorithm used in artificial intelligence designed to mimic the behavior of a system using mathematical models based on a dataset [10]. They find applications in various domains, such as data classification and pattern recognition, which are among the most commonly used [11]. As illustrated in Figure 5, an ANN is composed of multiple layers of neurons. Each neuron computes a linear combination of its input variables and then applies an activation function to produce its output. Activation functions introduce a nonlinear relation within the neurons. The ANN is able to capture complex nonlinear patterns. Examples of commonly used activation functions include hyperbolic tangents and Rectified Linear Unit (ReLU). The weights $\omega_{i}$ utilized for the linear combinations in each neuron are optimized to minimize the loss function.

**FIGURE 6:**
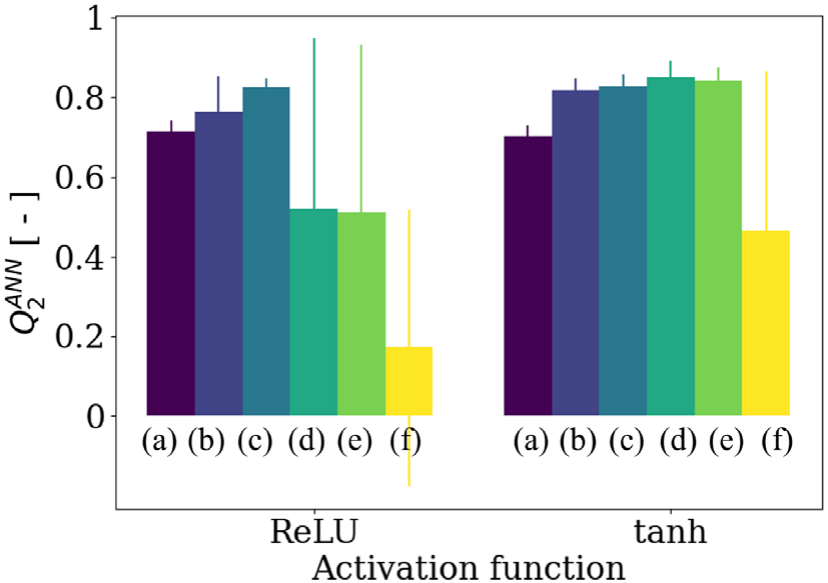
Effect of the activation function (ReLU or tanh) and the architecture of the ANN on the accuracy of the predictions, quantified with $Q_{2}$. The notations (a), (b), (c), (d), (e), and (f), respectively, correspond to the following architectures: $\left(8\right)$, $\left(8,4,2\right)$, $\left(16,8,4,2\right)$, $\left(8,8,8\right)$, $\left(16,16,16\right)$, $\left(64,32,16,8,4,2,1\right)$, and $\left(64,32,16,8,4,2\right)$. The error bars correspond to the standard deviation between the five predictivity factors obtained with 5‐fold cross‐validation. The predictivity factor $Q_{2}^{\mathrm{ANN}}$ is maximized when using a hyperbolic tangent activation function is used on an ANN with architecture (d), resulting in $Q_{2}^{\mathrm{ANN}}=0.85$.

In this article, we employed the ScikitLearn Python machine learning library [11] to conduct a grid search aimed at determining the optimal configuration of the neural network (number of layers, number of neurons per layer, and the activation function) that would yield the most accurate predictions. We compared two of the most commonly used activation functions: the ReLU, defined as $g\left(z\right)=z$ if z>0 and 0 otherwise, and the hyperbolic tangent $g\left(z\right)=\tanh\left(z\right)$. Several architectures were then tested. They are denoted as $\left(n_{1},n_{2},\ldots ,n_{N}\right)$, where $n_{i},i\in \left\{1,2,\ldots ,N\right\}$ is the number of neurons contained in the $i^{\mathrm{th}}$ layer. The tested architectures included $\left(8\right)$, $\left(8,4,2\right)$, $\left(16,8,4,2\right)$, $\left(8,8,8\right)$, $\left(16,16,16\right)$, $\left(64,32,16,8,4,2,1\right)$, and $\left(64,32,16,8,4,2\right)$. Other neural network hyperparameters, such as the solver algorithm or the optimization technique, were set according to the default parameters implemented in the library. The $Q_{2}$ values were evaluated using 5‐fold cross‐validation. Figure 6 illustrates how the activation function and the architecture of the ANN affect $Q_{2}$. Neural networks utilizing a hyperbolic tangent activation function produced the most accurate predictions, regardless of the architecture.

**FIGURE 7:**
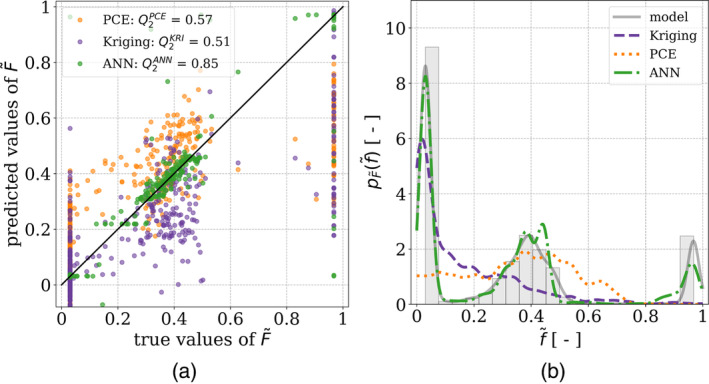
(a) Predicted vs. true values obtained with the ANN along with (b) a comparison of the PDFs obtained for the predictions of this dataset and that of the data from the model.

The predictions obtained using the specified ANN configuration are depicted in Figure 7. Furthermore, the predictivity factor obtained for these predictions is $Q_{2}^{\mathrm{ANN}}=0.85$, which is significantly better than those obtained using Kriging and PCE metamodels. One can also note that all the predictions fall within the range of the domain of definition of $\tilde{F}$, that is, $\left[0,1\right]$. Furthermore, the estimated PDF of $\tilde{F}$ based on the outputs of the ANN closely matches that obtained based on the data of the original model. Based on this result, the values of $\tilde{F}$ generated by the ANN built in this study can be used to conduct the sensitivity analysis.

## 3 Sensitivity Analysis

Sensitivity analysis is a field of computational mathematics that aims at determining the model input parameters that have the greatest influence on a quantity of interest (QoI) [12, 13]. It serves various purposes, including model simplifications or guiding research efforts, as their results can be used to optimize the resources necessary for experimental in vitro investigations. Studies such as [14, 15] demonstrate the applications of sensitivity analyses, particularly in biomechanics. There are three distinct types of sensitivity analysis: screening methods, local sensitivity analysis, and variance‐decomposition‐based methods [16, 17]. Screening and variance‐decomposition‐based methods are two branches of global sensitivity analysis, as described by [12]. Screening methods involve identifying unimportant input parameters using a limited number of model calls. On the other hand, local sensitivity analysis techniques involve assessing the impact of small input variations around a given point on the model's output. While this method can provide quick results with few data points, it may not perform well for nonlinear or nonmonotonic models. Variance‐decomposition‐based sensitivity analyses investigate the influence of input variability on the output by studying the contribution of each input to the output variance. This is accomplished by sweeping the entire domains of input parameter definitions. Global sensitivity analyses have been preferred over local techniques in this work due to the nonlinearities and likely nonmonotonicities of the model's response (refer to Figure 1). For a detailed analysis of these methods, please consult [12, 18, 19] and references therein. According to [20], several variance‐decomposition‐based sensitivity analysis techniques can be used in the case of nonmonotonic models. However, only two of them provide information on the effects for all orders. This includes the effect of a variable alone (first order) as well as its interactions with one (second order) or all (total) other parameters. These are the Sobol indices, and their estimation is also referred to as analysis of variance (ANOVA) in the literature [21]. The Sobol sensitivity indices are used to estimate the contribution of an input random variable on the variance of the output. The first order and total Sobol indices for a variable $X_{i}$ are defined in Equation 3a, where $X_{i},i\in \left\{1,2,\ldots ,M\right\}$ is the set of M input variables, Y represents an output QoI, and E denotes the expectation. The first‐order index $S_{i}$ estimates the part of the variance of Y due to $X_{i}$ alone. Moreover, the total index ${\mathrm{ST}}_{i}\frac{1}{2}$ accounts for the effect of interactions with other variables $X_{j,j\neq i}$.

(3a) $$S_{i}=\frac{\mathrm{Var}\left[E\left[Y|X_{i}\right]\right]}{\mathrm{Var}\left[Y\right]},$$

(3b) $${\mathrm{ST}}_{i}=1-\frac{\mathrm{Var}\left[E\left[Y|X_{1},\ldots ,X_{i-1},X_{i+1},\ldots X_{M}\right]\right]}{\mathrm{Var}\left[Y\right]},$$

in which E denotes the expectation.

**FIGURE 8:**
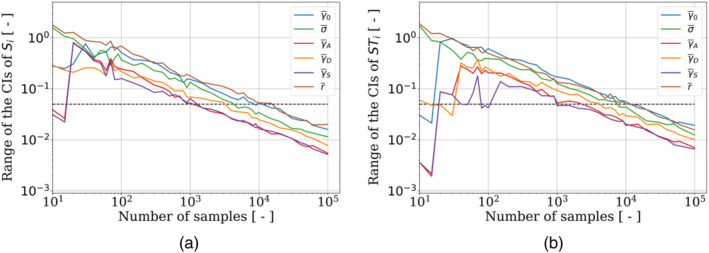
Range of the 95% confidence intervals of the (a) first and (b) total Sobol indices, in terms of the number of estimations of the metamodel, computed with the Mauntz–Kucherenko algorithm. The dashed lines represent the threshold of 0.05.

Several approaches are available to numerically compute the Sobol indices, which estimate different terms involving the evaluation of the variance in Equation (3) [22]. For instance, Saltelli [23], Mauntz‐Kucherenko [24], Martinez [25], and Jansen [26] have developed techniques to estimate these terms. The computation of the Sobol indices depends on the number of samples that are used to estimate the variance. It is therefore essential to ensure a sufficient sample size. In this specific case, when the QoI (i.e., the Sobol indices) can be very close to zero (indicating a non‐influential parameter), the conventional convergence techniques based on the study of a normalized gradient of the QoI cannot be applied. Alternative criteria for assessing the convergence of sensitivity indices have therefore been proposed in the literature. One such criterion, proposed by [27], involves evaluating the variability of the sum of the sensitivity indices generated using two samples of different sizes. One drawback of this criterion is that it does not examine the convergence of each sensitivity index individually. In addition, Herman et al. [28] considered a threshold for the percentage of the sensitivity index of the most influential input parameter, which does not investigate the convergence of each index either. Then, Sarrazin et al. [29] proposed a convergence study based on the range of the 95% confidence intervals (CIs) of the indices. They defined that the convergence is reached when the latter is smaller than 0.05. Note that the value of the convergence threshold should be adjusted according to the nature of the problem and available data. The criterion must be fulfilled for all the indices. Figure 8 displays the range of the 95% CIs of the Sobol indices on a log‐log scale, based on the number of samples used for their approximation using the Mauntz–Kucherenko algorithm. According to these graphs, a minimum of $2\times 10^{4}$ samples is required for the Sobol indices to converge. The latter, approximated with $10^{5}$ samples, that is, enough for the convergence to be ensured, are represented in Table 1.

**TABLE 1:** First and total Sobol indices, computed using $10^{5}$ estimations of the ANN.

Index	Parameter	Sobol indices
$S_{i}$	${\bar{\gamma }}_{0}$	0.10
	$\bar{\sigma }$	0.02
	${\bar{\gamma }}_{A}$	0.01
	${\bar{\gamma }}_{D}$	0.03
	${\bar{\gamma }}_{S}$	< 0.01
	$\bar{r}$	0.48
${\mathrm{ST}}_{i}$	${\bar{\gamma }}_{0}$	0.36
	$\bar{\sigma }$	0.16
	${\bar{\gamma }}_{A}$	0.07
	${\bar{\gamma }}_{D}$	0.15
	${\bar{\gamma }}_{S}$	0.05
	$\bar{r}$	0.83

According to the Sobol indices, the aspect ratio of the NP, $\bar{r}$, is the most important parameter, with ${\mathrm{ST}}_{\bar{r}}=0.83$ and $S_{\bar{r}}=0.48$. The initial adhesion force between the NP and the cell membrane, ${\bar{\gamma }}_{0}$, ranks as the second most influential parameter, exhibiting ${\mathrm{ST}}_{{\bar{\gamma }}_{0}}=0.36$ and $S_{{\bar{\gamma }}_{0}}=0.1$. This is followed by the membrane tension $\bar{\sigma }$ (${\mathrm{ST}}_{\bar{\sigma }}=0.16$ and $S_{\bar{\sigma }}=0.02$) and the delay of the transition of adhesion ${\bar{\gamma }}_{D}$ (${\mathrm{ST}}_{{\bar{\gamma }}_{D}}=0.15$ and $S_{{\bar{\gamma }}_{D}}=0.03$). The relatively small first‐order indices but large total indices for these last parameters indicate that their primary contribution to the variance of the model's output arises from interactions with other, more influential parameters, suggesting their relevance in the study. The remaining parameters have a negligible influence, as indicated by their total and first‐order indices being close to zero.

## 4 Discussion

The primary goal of this article is to analyze the impact of parameters on the model to account for the adaptation of adhesion between the membrane and the NP during the wrapping process. A sensitivity analysis was conducted to measure the effect of the model parameters on the wrapping degree at equilibrium $\tilde{f}$. However, recalling that the context of this work is to discriminate the entry of NPs into cells based on their mechanical properties for targeted cancer therapy applications, it is more relevant to focus on the capacity of the NP to enter the cell or not. In this case, it is necessary to consider not only $\tilde{f}$ but also the distance between the two free sides of the membrane. This is required for the merging of the membrane to ensure that the NP cannot be expelled by the cell [1]. Therefore, instead of using a continuous variable $\tilde{f}$, a binary variable indicating whether the merging occurred or not could have been used as the quantity of interest (QoI) for the sensitivity analysis. The data for this variable could have been generated using a classification learning algorithm. This article did not explore this approach, but it requires further investigation. Furthermore, based on the regression approach used in this study, the ANN was able to provide accurate predictions for the model. However, it is important to note that prediction errors may still occur ($Q_{2}=0.85$), and the impact of these uncertainties on the Sobol indices predictions has not been quantified. The predictions based on ANN could have been improved by investigating additional hyperparameters. Indeed, only two types of activation functional were used in this paper, although it would have been possible to assign one type of activation function per neuron. Experimenting with additional solvers and optimization techniques might also lead to more accurate predictions. However, the learning model's objective was to generate data while minimizing computational costs. Therefore, it would not have been reasonable to spend considerable resources optimizing the design and hyperparameters of the ANN.

## 5 Conclusion

The article presents the significant role of machine learning, and particularly ANNs, in overcoming computational cost issues. They accurately emulate the behavior of a model with a near‐to‐zero computation time. The study shows that ANNs can replicate the model of cellular uptake of undeformable elliptic NPs while also considering the adhesive response of the cellular membrane to the interaction with the NPs. This surrogate model outperformed Kriging or PCE in terms of accuracy, with $Q_{2}^{\mathrm{ANN}}=0.85$, compared to $Q_{2}^{\mathrm{PCE}}=0.57$ and $Q_{2}^{\mathrm{KRI}}=0.51$. The approximations generated by ANN could therefore be used to perform a sensitivity analysis. The analysis shows that the aspect ratio of an NP is the most important parameter for cellular uptake, followed by the initial adhesion between the NP and the cell membrane, the membrane tension, and the delay before the increase of adhesion. The slope and amplitude of the variation of adhesion between the NP and the membrane do not appear to affect the equilibrium wrapping degree of the NP by the membrane. Identifying these parameters is crucial, as it reduces the number of variable parameters for experimental studies. Indeed, if the hypotheses used to build the model presented in this article are respected, the amplitude and ratio of the mechanical adaptation of the adhesion between the NP and the membrane, along with the membrane tension, can be disregarded.

**Nomenclature**
γ
: adhesion
$\bar{\gamma }$
: adimensional adhesion
${\bar{\gamma }}_{0}$
: adimensional initial adhesion
$\bar{\sigma }$
: adimensional tension
$\bar{r}$
: aspect ratio of the NP
κ
: bending rigidity
${\bar{\gamma }}_{S}$
: curvature parameter for the transition of adhesion
${\bar{\gamma }}_{D}$
: delay of the transition of adhesion
N
: number of samples
$Q_{2}$
: predictivity factor
a
: radius of the NP
$\bar{\Gamma }$
: random adimensional adhesion
${\bar{\Gamma }}_{0}$
: random adimensional initial adhesion
$\bar{\sum }$
: random adimensional tension
${\bar{\Gamma }}_{S}$
: random curvature parameter for the transition of adhesion
${\bar{\Gamma }}_{D}$
: random delay of the transition of adhesion
${\bar{\Gamma }}_{A}$
: random ratio between final and initial adimensional adhesion
${\bar{\gamma }}_{A}$
: ratio between final and initial adimensional adhesion
$S_{i}$
: Sobol first order index with respect to input parameter i
σ
: tension
${\mathrm{ST}}_{i}$
: total Sobol index with respect to input parameter i
f
: wrapping degree
ANN: artificial neural network
CI: confidence interval
MC: Monte Carlo
NP: nanoparticle
PCE: polynomial chaos expansion

## Conflicts of Interest

The authors declare no conflicts of interest.

## Supporting information


**Data S1** Supporting Information.

## Data Availability

The data that support the findings of this study are openly available in Zenodo at https://zenodo.org/doi/10.5281/zenodo.13734428, reference number 13734429.
