# Supplementary figures and images for: PREPRINT Machine Learning for the Sensitivity Analysis of a Model of the Cellular Uptake of Nanoparticles for the Treatment of Cancer
Source: Int J Numer Method Biomed Eng. 2024 Oct 29;40(12):e3878. doi: 10.1002/cnm.3878 (PMC11618229; doi:10.1002/cnm.3878)

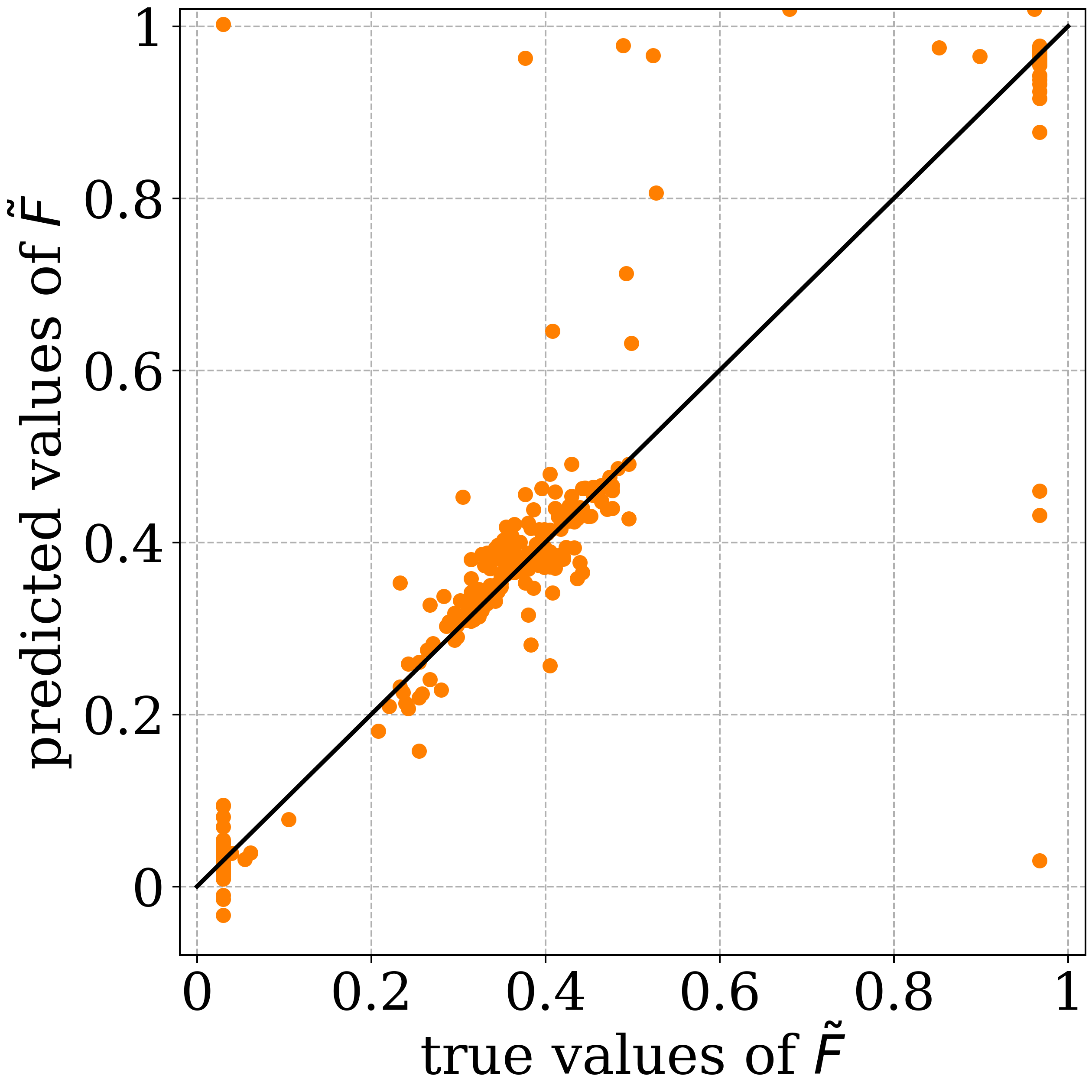

Supplement: Supplementary file 1 — Data S1 Supporting Information. [file CNM-40-e3878-s001.zip › ANN-sensitivity-adaptive-uptake-of-elliptic-nanoparticles/uptake/ANN_mechanoadaptation_vs_passive_elliptic_new_settings_article-v2_360.png]

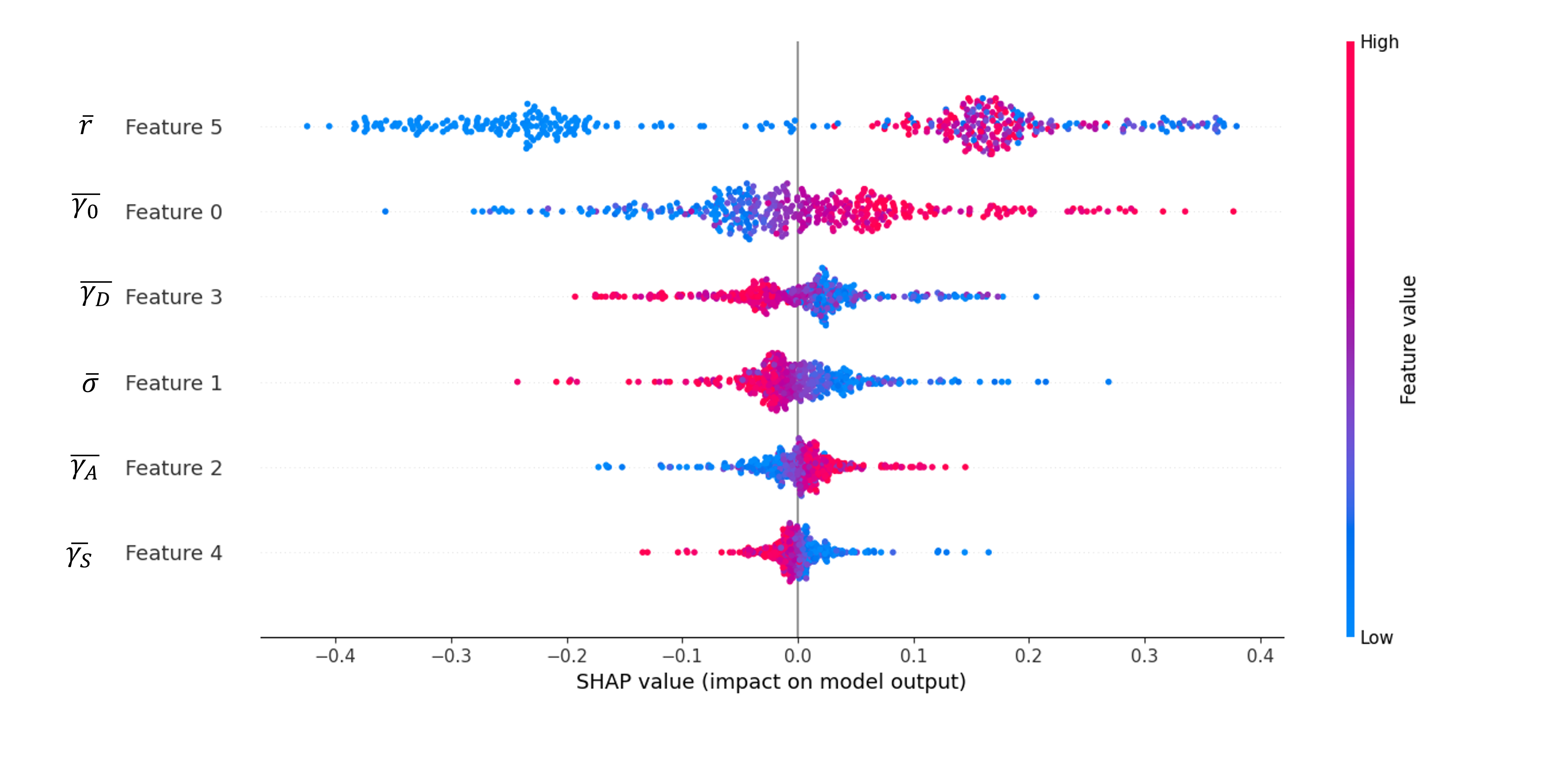

Supplement: Supplementary file 1 — Data S1 Supporting Information. [file CNM-40-e3878-s001.zip › ANN-sensitivity-adaptive-uptake-of-elliptic-nanoparticles/uptake/Shapley_Values.png]
